# Inhaled carbon monoxide or nebulized sodium nitrite protect against hemorrhagic shock-induced mitochondrial dysfunction

**DOI:** 10.1186/cc12158

**Published:** 2013-03-19

**Authors:** H Gomez, D Escobar, B Ataya, L Gordon, O Ogundele, M Pinsky, S Shiva, B Zuckerbraun

**Affiliations:** 1 University of Pittsburgh, PA, USA

## Introduction

Shock induces mitochondrial damage, which can lead to tissue injury and inflammation. Resuscitative adjuncts to limit mitochondrial injury may be effective to reduce tissue injury and protect against the sequelae of hemorrhagic shock (HS). Others and we have demonstrated the protective effects of inhaled carbon monoxide (CO) or nebulized sodium nitrite (NaNO_2_) in models of HS. Our aim was to test the hypothesis that CO and NaNO_2 _protect against hemorrhagic shock-induced tissue injury/inflammation by limiting mitochondrial damage and preventing bioenergetic failure.

## Methods

Twenty anesthetized female Yorkshire pigs were subjected to severe hemorrhage until unable to compensate or 90 minutes, and were then resuscitated with volume/pressors. Muscle and platelet samples were obtained at baseline (BL) and 2 hours after resuscitation (EndObs). Animals were randomized to: standard of care (HSR, *n = *5); HSR+CO (CO; 250 ppm×30 minutes, *n = *6); or HSR+NaNO_2 _(NaNO_2_; 11 mg in PBS×30 minutes, *n = *6), and sham (*n = *3). CO or NaNO_2 _were initiated ~30 minutes before resuscitation. Primary endpoints were changes in muscle and platelet mitochondrial respiration between BL and EndObs, quantified by muscle respiratory control ratio (RCR, traditional respirometry), and by the change in proton-leak respiration (PLR) and mitochondrial reserve capacity in platelets. Secondary endpoint was mortality at EndObs.

## Results

Skeletal muscle RCR decreased in the HSR group (*P *= 0.04) but not in sham. Decrease in RCR was primarily due to decreased ADP-dependent respiration, without change in state 4 respiration. HSR also resulted in platelet mitochondrial dysfunction as demonstrated by increased PLR and decreased reserve capacity. This correlated with increased platelet activation (%CD62P+ by flow cytometry) in HSR. CO or NaNO_2 _treatment prevented these deleterious changes in both muscle and platelet mitochondrial respiration, as well as limited HSR-induced platelet activation. CO treatment also improved reserve capacity compared with baseline. Mortality was higher in HSR than in CO or NaNO_2 _(80 vs. 33 and 33%, respectively).

## Conclusion

In severe HS, mitochondrial injury in platelets and muscle was limited by CO or NaNO_2_. Although not powered for a secondary endpoint, mortality was double in HSR versus adjunctive therapies. This suggests that CO and NaNO_2 _may protect mitochondrial function by maintaining ATP-coupled respiration and reserve capacity, and that this may confer a survival advantage. However, further investigations are required.

